# Hybrid LSTM Self-Attention Mechanism Model for Forecasting the Reform of Scientific Research in Morocco

**DOI:** 10.1155/2021/6689204

**Published:** 2021-05-25

**Authors:** Asmaa Fahim, Qingmei Tan, Mouna Mazzi, Md Sahabuddin, Bushra Naz, Sibghat Ullah Bazai

**Affiliations:** ^1^College of Economics & Management, Nanjing University of Aeronautics & Astronautics, Nanjing 210016, China; ^2^Mohammed V University, Rabat, Morocco; ^3^Department of Computer Systems Engineering, Mehran University of Engineering and Technology, Jamshoro, Kotri, Sindh 76062, Pakistan; ^4^Department of Computer Engineering, Balochistan University of Information Technology, Engineering and Management Sciences, Quetta, Balochistan 87300, Pakistan

## Abstract

Education is the cultivation of people to promote and guarantee the development of society. Education reforms can play a vital role in the development of a country. However, it is crucial to continually monitor the educational model's performance by forecasting the outcome's progress. Machine learning-based models are currently a hot topic in improving the forecasting research area. Forecasting models can help to analyse the impact of future outcomes by showing yearly trends. For this study, we developed a hybrid, forecasting time-series model by long short-term memory (LSTM) network and self-attention mechanism (SAM) to monitor Morocco's educational reform. We analysed six universities' performance and provided a prediction model to evaluate the best-performing university's performance after implementing the latest reform, i.e., from 2015–2030. We forecasted the six universities' research outcomes and tested our proposed methodology's accuracy against other time-series models. Results show that our model performs better for predicting research outcomes. The percentage increase in university performance after nine years is discussed to help predict the best-performing university. Our proposed algorithm accuracy and performance are better than other algorithms like LSTM and RNN.

## 1. Introduction

The development of science and technology plays an essential role in strengthening a country and enriching its people. Recently, African scientific research has attracted the attention of many countries and international organisations [1]. The United Nations Educational, Scientific, and Cultural Organization (UNESCO) believes that science and technology guarantee sustainable development [2]. The use of science and technology to understand and transform the world is the primary means of solving current economic, social, and environmental problems. All countries globally should cooperate and assist one another to jointly research and develop science and technology. UNESCO supports countries in the investment of science, technology, and innovation (STI) and the formulation of related policies. It also provides countries with assistance in reforming the scientific system and their scientific and technological evaluation [3].

**Figure 1:**
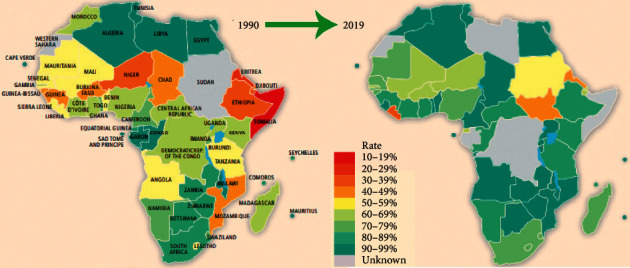
Africa education system net primary education enrollment rate of students from 1990 to 2019.

Similarly, the Organization for Economic Cooperation and Development (OECD) supports and encourages countries to aid scientific research in Africa. It recommends that donor countries create regional and Africa-wide scientific and technological networks to support further the scientific and technological systems and higher education of African countries [4]. The OECD believes that assistance to Africa should go beyond limited validity and extend to broaden development validity. Sponsoring the development of scientific research in African countries and supporting the creation of an African scientific research platform are important strategies for promoting economic transformation. Also, many international organisations and institutions support the development of scientific research in Africa [5]. For example, the Institute for Development Research (IRD) in France has been paying attention to and researching Africa for 65 years. The institute advocates combining the needs of African countries to support the development of local scientific research. Changes in the last 30 years have greatly improved education systems, as shown in Figure 1.

At the end of the twentieth century, Morocco's rapid scientific research and development reached a climax [6]. During the same period, South Africa and Egypt were still the leaders in scientific research in the African region. However, their development was slow compared with that of the subsequent latest reforms. At the same time, Nigeria, which was initially third in Africa, rapidly reduced research capacity and output, while Kenya maintained its development level. Africa's momentum is slightly less than that of Maghreb countries [7]. During the same period, the crisis of universities in other African countries limited scientific research development. Since the start of the twenty-first century, Morocco has continuously promoted scientific research reforms to encourage research development. Morocco successfully launched its first scientific satellite in 2001 and joined the European Union's Galileo global satellite navigation system in 2005. It became the first African and Arab country to join the program and the fifth non-EU country to participate in the program after China, India, Israel, and Ukraine [8]. These scientific research's achievements have played an important role in Morocco's economic development and the improvement of its international competitiveness.

Historically, Moroccan scientists have made significant contributions to the field of natural sciences. Moroccan-born Serge Haroche was jointly awarded the Nobel Prize for Physics in 2012 with a USA physicist [9]. Haroche's work in quantum physics was recognised for its ground-breaking experimental methods that enable measuring and manipulating individual quantum systems. Baruj Benacerraf received the 1980 Nobel Prize in Medicine for discoveries concerning genetically determined cell surface structures that regulate immunological reactions. Also, Adnane Remmal is a Moroccan biology professor known for discovering the antimicrobial activity of essential oils. Morocco's increase in research and innovation progress was made possible by the ongoing changes in the educational model brought on by new reforms. Morocco has implemented many educational reforms, which we discuss in the next section, but it is imperative to monitor those reforms' performance. The development of a forecasting model for performance prediction of educational reform is necessary. Currently, many forecasting models are being used in higher education that is based on time-series data. They include trend extrapolation, population dispersion prediction models, forecasting methods, grey prediction methods, linear regression models, distributed lag models, logistic models, artificial neural network methods, and others. Time-series analysis can be used for the trend analysis of time-series data [10, 11]. Time-series data are data that are arranged according to a series of periods or intervals. Time-series analysis involves testing linear or nonlinear relationships among dependent variables. Many linear and nonlinear approaches have been proposed for forecasting in various time-series studies [12]. Linear approaches include exponential smoothing [13] and regression-based models, such as autoregressive integrated moving average (ARIMA) [14, 15] trigonometric seasonality, Box-Cox transformation with ARIMA residuals, and trend and seasonal components (TBATS) [16–18].

In recent years, with the continuous in-depth research of machine learning by researchers, artificial neural networks (ANNs), deep learning (DL), reinforcement learning (RL), migration learning (transfer learning), and other novel algorithms are developed, so time-series analysis and forecasting methods have been in-depth research and rapid development. Machine learning applications are currently implemented in almost all field of study; in field of medical, it is used for ultrasound recognition [19] and classification of heart diseases [20]. Based on supervised learning, model decomposition, seasonal adjustment, particle swarm optimisation (PSO), and least square support vector machine (LSSVM) models are helpful when combined for forecasting purposes like Chen et al. used the combined forecasting method (ESPLSSVM) to predict power load data and proposed a dimensional-weighted residual long short-term traffic prediction [21]. Based on the system architecture of Tensorflow, combining RNN and LSTM, GRU network can be helpful in forecasting. Machine learning methods using CNN have many further applications in daily life as Pu et al. developed a traffic forecasting system based on human behaviour [22]. Huang et al. developed a health recommendation system using improved KNN methods based on label-based learning [23]. Han et al. developed tag-based methods to classify movies recommendation using KNN [24]. Bhatti et al. used feature-based pattern extraction for medical diseases using CNN [25].

Nevertheless, it is found that the RNN and LSTM are very suitable for analysing and studying time-series data but with these shortcomings:

1. RNN network has gradient explosion and gradient disappearing problems.
2. RNN gradient problems have been solved to a certain extent in LSTM and its variants, but it is still not enough. It can handle sequences of 100 orders of magnitude but is still complex for sequences of 1,000 orders of magnitude or longer.
3. Time-consuming calculation: each series of LSTM means that there are multilayer perceptron (MLP). If the period of LSTM is extensive and the network is deep, the amount of calculation will be significant and time-consuming [26].

Therefore, this article propose a hybrid method which is combination of LSTM with attention mechanism and found that this method effectively predicts time-series data and helps solve the above problems. This study focuses on developing a new prediction model to help monitor research in the higher education system that can be extended to other higher education components, i.e., student enrollment and faculty development. The main contributions of this research are as follows:

1. We developed a new forecasting algorithm, SAM-LSTM, which is a fusion method of self-attention mechanism (SAM) and long short-term memory network (LSTM). This model's characteristic is that each data input can be assigned more detailed and different weights to prevent missing essential and critical information. It thereby improves processing results like accurate judgment and reduces space consumption problems caused by data storage.
2. Comparison of universities' research outcomes for the coming years, identifying the best-performing university through current educational reforms.
3. Comparison of old and new education reforms by forecasting both the trend of reforms and predicting best-performing reform.
4. Comparison of a new forecasting algorithm with other RNN and LSTM models.

This paper's structure is divided as follows: in the literature review, we introduce Moroccan educational reforms to help predict the performance of new educational reforms. Furthermore, we present the latest forecasting models for performance monitoring that have been developed in the education sector. In the next section, we propose our new time-series forecasting model. It is followed by a description of our experimental analysis of research performance in Moroccan universities with forecasts. Finally, we present our conclusions and recommendations for future directions.

## 2. Literature Review

### 2.1. Morocco Education Reform

Long before the West colonised Morocco, Moroccan higher education had a certain level of development. To overcome colonialism's influence and develop its higher education after independence, Morocco has carried out many reforms in its higher education system. At the end of the twentieth century, in the face of continued economic globalisation and increasingly fierce international economic competition and the profound impact of economic globalization on education, the Moroccan government carried out comprehensive educational reform. In 1999, the government created the National Education and Training Charter and implemented ten years of educational reform beginning in 2000 [27].

The National Education and Training Charter is a programmatic document formulated by the Moroccan government for twenty-first century educational reform. The charter includes four aspects of higher education reform: (a) primary objectives requiring higher education to adapt to the development of society and the market economy; (b) decentralization of higher education management; (c) implementation of university autonomy; and (d) improvement in education and teaching [28, 29].

In 2008, when the National Education and Training Regulations had been in effect for nearly ten years, the Moroccan government noticed that there was still a large gap between the achievements of the educational reforms and the initial goals. Today, there are still many difficulties with the reform goals set in the constitution. To consolidate educational reform achievements and realise the original goals of that reform, the Moroccan government established the National Education Emergency Support Program in 2009 with plans to implement a four-year (starting from 2009–2010) education reform program [30]. As it relates to higher education, the contents of the National Education Emergency Funding Program include the following:

1. Further increasing the financial investment in higher education
2. Promoting additional decentralisation of power in higher education management
3. Striving to improve the quality of higher education
4. Upgrading scientific research and innovation capabilities in universities

Since 2000, enrollment numbers in Moroccan higher education have shown a continuous growth trend. However, like many developing countries, the expansion of Moroccan higher education has simultaneously led to a decline in education quality. According to data provided by the 2014-2015 Global Competitiveness Report and 144 countries included in the evaluation, Morocco ranked 102nd in the overall education quality and 104th in higher education quality and training systems [31]. An important reason for the low quality of Moroccan higher education is that many schools use financial subsidies primarily to expand enrollment rather than to improve instruction quality. The lack of competition between Moroccan universities means that some institutions are content with low standards and are unwilling to make changes. More than 85% of budget funding designated for higher education development is used for current expenditure but only 0.5% for research and 1% for staff training. The massive expenditure on education has had an unsatisfactory return [32]. The Moroccan Supreme Council for Education, Training, and Scientific Research has established a roadmap for reforming Moroccan educational and training systems for 2015–2030 [33]. This roadmap focuses on resolving students' poor academic achievement, improving scientific research and innovation, integrating and teaching foreign languages, reducing school dropouts, and increasing connections between education and the demands of the job market. Our study focuses on forecasting the research outcomes for Moroccan universities' latest trends with a view to current reform.

### 2.2. Forecasting in the Education System

Currently, forecasting the latest education trends is a hot topic of discussion in the education sector. Griffiths highlighted the risk factors for quality assurance in higher education in his study using a machine learning algorithm model for neural networking [34, 35]. Another study shows the forecasting of student enrollments in higher education institutions (HEIs) using the ARIMA model as a technique [36, 37]. Al-Haddad et al.'s study revealed the graduation rates of university students by using a multiple-regression model. Analysis of variance (ANOVA) is also helpful in predicting a skilled workforce's needs as a factor in improving higher education quality [38]. However, the methodology of the regression model has certain limitations. It can only be used for data subject to the linear process [39]. The time-series analysis method is also a kind of regression method and does not consider the role of explanatory variables based on population theory or economic theory. Instead, it uses the extrapolation mechanism to describe and predict the change in the time series based on the variable's changing law. Many authors have used various time-series models such as ARIMA, Prophet, and Grey (1, 1), to name a few, to forecast educational system performance [40]. Shantini and Suriya [41] studied the educational system's impact on economic development and developed a forecasting model using ARIMA. Ghosh [42] developed a hybrid model for an educational system using ARIMA with ANN to predict education system expenditure. The fractional grey Bernoulli model with a differential evolution algorithm helps forecast educational-sector investment [43].

Singh and Srivastava believed that existing ANN methods cannot provide encouraging results. Deep learning for stock prediction is introduced and its performance is evaluated based on Nasdaq's Google stock price multimedia data (charts) [44]. The (2D) PCA + deep neural network (DNN) combination method is compared with the existing method 2D PCA + radial basis function neural network (RBFNN). It is found that this method has better performance than the existing RBFNN method, and the accuracy of the hit rate with a batch size of 20 is improved by 4.8%. The model results were also compared with the RNN, and it was found that the accuracy of the hit rate increased by 15.6%. The correlation coefficient between DNN's actual income and predicted income is 17.1% higher than that of RBFNN and 43.4% higher than that of RNN. Therefore, our research paper proposed a new direction of the LSTM improved algorithm.

## 3. Preliminary and Proposed Forecasting Model

This section mainly introduces the long short-term memory neural network and the attention mechanism and the improved LSTM method model composed of the two. It then analyses the short-term memory network and the advantages of the attention model and then combines the two models to overcome disadvantages.

### 3.1. LSTM Neural Network

In 2012, Sundermeyer and others jointly proposed the long short-term memory neural network (referred to as LSTM network) [45]. Its function is to solve the problem of RNN's inability to handle long-distance long-sequence dependence, and LSTM is an improvement of the RNN network structure [46]. The cyclic neural network is because the gradient disappears during the data sample training, which makes it impossible to process a long time series [47]. The gradient disappears because the derivative index becomes smaller during the calculation, which leads to the weakening of the RNN's perception ability.

Every scientific researcher worked hard to study and finally discovered the characteristics of insufficient gradient disappearance of the optimised recurrent neural network. The state calculation formula of RNN is as follows:

(1) $$\begin{array}{c} S_{t}=f\left(S_{t-1},X_{t}\right). \end{array}$$

The chain derivation rule adopts the gradient derivation and then becomes the multiplication form, which will cause the multiplication result to decrease rapidly under the sigmoid1 condition. In response to this problem, researchers changed their research thinking and adopted a cumulative method as shown in the following formula:

(2) $$\begin{array}{c} S_{t}=\sum_{\tau -1}^{t}\Delta S\tau . \end{array}$$

Furthermore, its calculated derivative is also in the additive form so that no gradient disappears. The proposed LSTM uses the cumulative form to increase the gate structure, and the network becomes more complicated. Next, the internal structure is explained.

**Figure 2:**
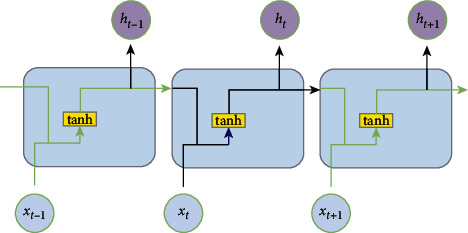
RNN model.

In Figure 2, an expanded diagram of the RNN model is shown, which contains multiple connections of recurrent layers, but the network structure is relatively simple. The recurrent layer only contains the tanh layer, as shown in Figure 2.

**Figure 3:**
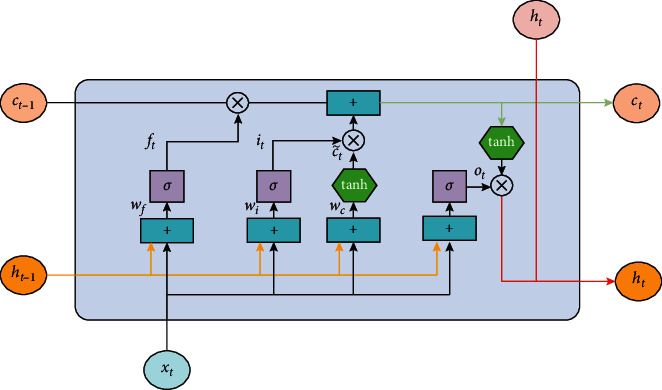
LSTM different gates and basic architecture.

However, it is proposed that the repetitive module of the LSTM network is much more complicated than the RNN repetitive module. It mainly adds three gate settings: forget gate, input gate, and output gate. The forget gate's function determines how much previous unit state information is retained in the current unit state. The input gate's function determines how much current moment information is retained in the current unit state. The output gate's function determines the output quantity of the state at the current moment. The long short-term memory neural network diagram is shown in Figure 3.

(1) *Forgotten Gate.* First, remember that the forget gate's function controls the proportion of cells selectively forgetting information. Its input is the output *h*_*t*−1_ at the previous time *t* − 1 and the input *X*_*t*_ at the current time *t*. After processing by sigmoid, it will be 0 to the value of 1: 1 means all reserved and 0 means all forgotten, so we have
  (3) $$\begin{array}{c} f_{t}=\sigma \left(W_{f}h_{t-1}+U_{f}X_{t}+b_{f}\right). \end{array}$$
In formula (3), *W*_*f*_ and *U*_*f*_, respectively, represent the weight matrix of the forget gate and *b*_*f*_ represents the bias term of the forget gate. *σ* is the sigmoid function.
(2) *Input Gate*. The input gate is followed by the function of the input gate to calculate which information is stored in the state unit.
The first part of the expression is as follows:
  (4) $$\begin{array}{c} i_{t}=\sigma \left(W_{i}h_{t-1}+U_{i}X_{t}+b_{i}\right). \end{array}$$
This part's function can be expressed as the final determination of how much current input information can be saved to the state.
The second part of the expression is as follows:
  (5) $$\begin{array}{c} \tilde{c_{t}}=\text{tanh}\left(W_{c}h_{t-1}+U_{c}X_{t}+b_{c}\right). \end{array}$$
Among them, *W*_*i*_ and *W*_*c*_ represent the input layer's weight, *b*_*i*_ and *b*_*c*_ represent the input layer's bias, tanh as the activation function, and *U*_*i*_ and *U*_*c*_ are the input layer's weight matrix. This part can mean adding new information generated by the current input to the state. The first and second results are combined to build new memories.
(3)   *Cell State.* The cell state *C*_*t* _ at the current moment is the product of the input of the forget gate and the state of the previous moment plus the product of the two parts of the input gate, that is, as follows:
  (6) $$\begin{array}{c} C_{t}=f_{t}{}^{*}C_{t-1}+i_{t}{}^{*}\tilde{c_{t}}. \end{array}$$
In formula (6), *C*_*t*−1_ represents the cell state at the previous moment and *C*_*t*_ represents the cell state value at the current moment.
(4)   *Output Gate.* The output gate plays a function in determining how much information is output. First, understand which information is to be output through the sigmoid function and then multiply the output information by the current unit state and use the tanh function value to output the following equations:

(7) $$\begin{array}{c} o_{t}=\sigma \left(W_{o}h_{t-1}+U_{o}X_{t}+b_{o}\right), \end{array}$$

(8) $$\begin{array}{c} h_{t}=o_{t}{}^{*}\;\tanh\left(C_{t}\right). \end{array}$$

Among them, *W*_*o*_ represents the output layer's weight and *b*_*o*_ represents the output layer's bias. The above is the working principle of long short-term memory neural networks.

### 3.2. Attention Mechanism

It can be seen from Figure 3 that the LSTM network does not solve all the problems. We still have a sequential path from the past unit to the current unit. This path is now more complicated because it has appendages and ignores the branches that belong to it. There is no doubt that LSTM and GRU (gated recurrent unit, a derivative of LSTM) and their derivatives can remember much long-term information. However, they can only remember a series of 100 orders of magnitude, not 1,000 orders of magnitude or more extended series. The RNN mentioned in the previous chapter has apparent shortcomings, and the computer hardware requirements are very high when training samples. When we do not need to train these networks too fast, it still requires a high level of resources. Running these models in the cloud also requires a high number of resources. This section introduces the attention mechanism (AM), which is the attention model proposed by Bahdanau et al. in 2014 [48] to overcome the above problems. Attention is mainly proposed to overcome the shortcomings of the RNN network and optimise the LSTM network. This model adds the Encoder + Decoder mechanism, which has a better effect.

The attention mechanism's essence is to help the model assign different weights to each sample data, extract essential information that affects data analysis and prediction, and make better estimates and judgments on the final result analysis. Also, the model will not consume a lot of computer memory, so this model is more commonly used.

**Figure 4:**
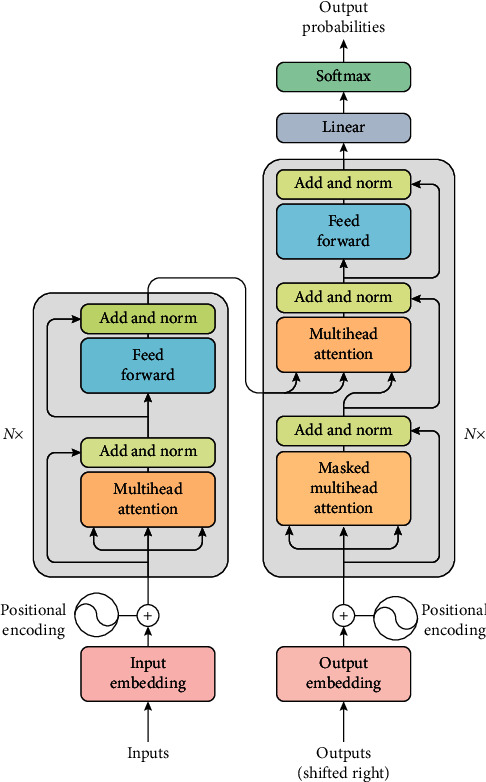
Self-attention mechanism.

The self-attention mechanism model contains three attention components, which are the encoder's self-attention and the attention that connects the encoder and decoder. The three attention modules (attention block) are all forms of multihead attention. The structure of the self-attention mechanism is shown in Figure 4.

The attention mechanism can reduce the influence of redundant and noisy data. The principle is to assign a reasonable weight ratio to the input data samples in training and learning.

**Figure 5:**
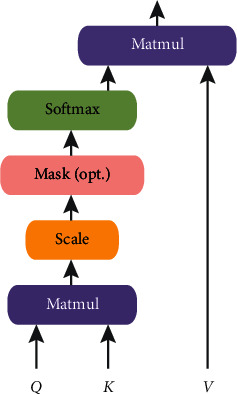
Scaled dot product attention.

In the attention mechanism, the main focus is a core module, which is the multihead attention in the self-attention mechanism. It uses multiple scaled dot product attention as the basic unit, which is then stacked in sequence. The logic diagram is shown in Figure 5. The input is all three elements of *Q* (query), *K* (key), and *V* (value). In the self-attention mechanism, *Q* = *K* = *V* takes the same value. The above attention can be described as mapping a set of query and key-value pairs to the output, where query, keys, values, and output are all vectors. The dimensions of query and keys are both *d*_*k*_, and the dimension of values is *d*_*v*_ (where *d*_*k*_ = *d*_*v*_ = *d*_(model/hour)=64_). The final output result calculation method uses values weighted summation, and the similarity functions of the query determine the weight of values, and keys are calculated together. That is, each encoder is composed of self-attention and a feedforward neural network. The data will first pass through the self-attention module to obtain a weighted feature vector *Z*. This *Z* is attention (*Q*, *K*, and *V*), as in the following formula:

(9) $$\begin{array}{c} \text{attention}\left(Q\text{,}K\text{,}V\right)=\text{ Softmax}\left(\frac{QK^{t}}{\sqrt{d_{k}}}\right). \end{array}$$

**Figure 6:**
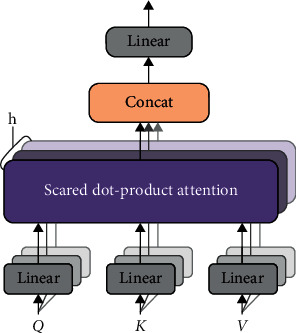
Multihead attention.

After mapping *Q*, *K*, and *V* through the parameter matrix (connect a fully connected layer to *Q*, *K*, and *V*, respectively), do self-attention, repeat this process *h* = 8 times, and finally concatenate all the results and then send it to a fully connected layer. Multihead attention is shown in Figure 6.

Self-attention is very different from the traditional attention mechanism. The traditional attention calculates attention based on the source's hidden state and target. The result is the dependency between each sequence of the source and each sequence of the target. However, self-attention is different. It is carried out on the source and target sides, respectively, and only differs from source input or target input.

Self-attention captures the dependency between the sequence of the source or target and then adds the self-attention from the source to the attention from the target and captures the word-to-word relationship between the source and target dependencies. Therefore, self-attention is better than the traditional attention mechanism. The traditional attention mechanism ignores the dependence between the source and target sequences. Compared with self-attention, it cannot only get the dependence between the source and target sequences because, simultaneously, it can also effectively obtain the dependence relationship between the source or target sequence itself.

### 3.3. Proposed SAM-LSTM Method

**Figure 7:**
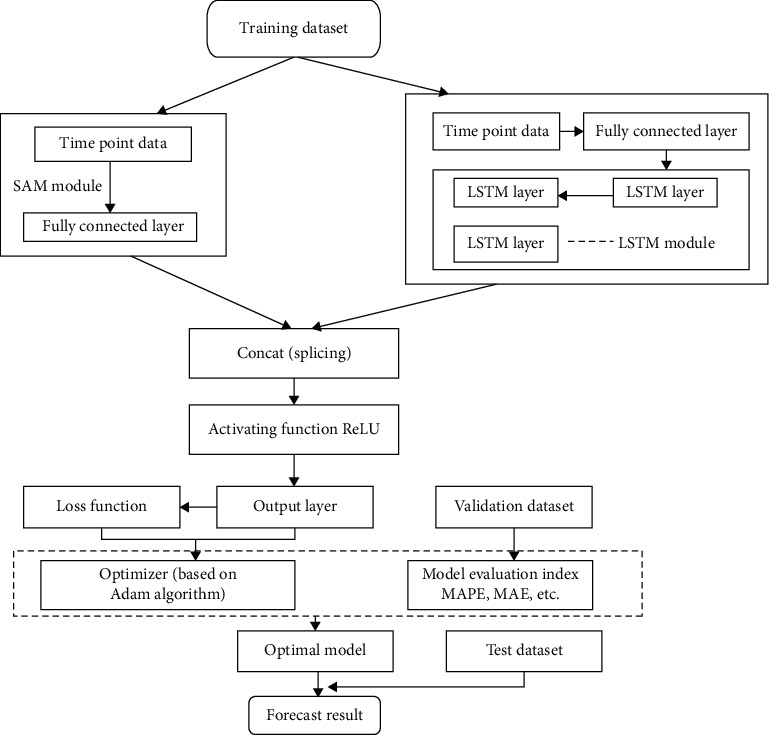
Proposed model workflow.

As described before, although long short-term memory networks can solve the long-term dependence of time series and solve the problem of gradient explosion and gradient disappearance of RNN to a certain extent, it is still not enough. It can handle sequences of 100 magnitudes, but it will still be tricky for 1,000 magnitudes or longer sequences. Therefore, this article comprehensively analyses the fusion of self-attention mechanism and LSTM neural network called SAM-LSTM method in response to the above problems. The network model of SAM-LSTM is shown in Figure 7.

The model consists of five components:

1. Input layer: input the time-series data of the model
2. Embedding layer: map each sequence to a low-dimensional size vector
3. LSTM layer: use LSTM to obtain advanced features from Step (2)
4. Attention layer: the self-attention mechanism generates a weight vector, weights the hidden state of all time steps (step), and focuses attention on the more important ones in the entire hidden state information sequence
5. Output layer: sequence-level feature vectors are finally used for time-series data analysis and prediction

The LSTM network and the attention mechanism are integrated into the network. Not only can the LSTM network make better use of the long-sequence data retention problem the self-attention mechanism can also rationally assign different weights to each part of the input data to extract more credible and helpful information to make better predictions on the forecast data.

## 4. Experiment and Results

### 4.1. Dataset Description

This study is based on research for outcomes prediction in Morocco. We selected six Moroccan universities' research outcomes from the world-renowned datasets Scopus and Science Direct [49]. The description of features for those data-collection libraries is shared below.

#### 4.1.1. Scopus Database

The Scopus database was launched by Elsevier in November 2004 and is currently the world's largest abstract and citation (A&I) database, covering 15,000 scientific, technical, and medical journals. The database contains more than 20,500 peer-reviewed publications from 5,000 publishing houses around the world (a complete collection of Elsevier, Springer/Kluwer, Nature, Science, American Chemical Society, Institute of Physics, American Physical Society, American Institute of Physics, and all journals by publishers such as Royal Society of Chemistry). It includes document types like journals, conference papers, book series, and patents, among many others–the yearly percentage increase in universities' research-publication performance after nine years as shown in Table 1.

The Science Direct database is Elsevier's core product and the world's largest full-text scientific literature database. It covers 21 disciplines in the fields of science, technology, and medicine (mathematics, physics, chemistry, astronomy, medicine, life sciences, business and economic management, computer science, engineering technology, energy science, environmental science, material science, social science, and many other disciplines). It provides retrieval and full-text download of more than 1,800 journals, of which 1,393 are included in SCI and 515 are included in EI.

Our dataset considers the research papers published by six universities: Al Akhawayn University Ifrane, Hassan II de Casablanca, Cadi Ayyad University, Université Ibnou Zohr, Université Mohammed V, and Ibn Tofail University. Research papers are considered for all the indexing services, i.e., SCI, SCIE, ESCI, EI, and conference publications. Research records from years 2000–2019 are extracted every year in different disciplines for time-series analysis.

### 4.2. Model Parameter Setting

Computer hardware configuration and machine learning network parameter settings are as described above. LSTM and SAM-LSTM prediction models are established based on SCOPUS and SPRINGER datasets, and Python is the software programming language used for experimental data. Python has a Keras package for deep neural network learning and a variety of data preprocessing and image drawing packages. It is simple to use and easy to learn, and beginners and research scholars use it widely. The specific network is set to the number of iterations (epoch) as 50/100 times, the activation function (activation) is rectified linear unit (ReLU), the number of samples selected for one training (batch_size) is 32, the optimiser is Adam = 0.0001, the hidden layer inside the size (unit) is 32, and the fully connected layer (dense) is 1.

### 4.3. Model Implementation Results

The purpose of SAM-LSTM is to determine the nature of the relationship between our residuals to provide our model with a certain degree of forecasting power. In the first instance, to conduct a time-series analysis, we must express our dataset in terms of logarithms. The time series is converted into a logarithmic format to smooth the volatility in the series.

ACF and PACF plots are generated to detect whether stationarity is present, and a Dickey–Fuller test is conducted to validate it. The time series is decomposed to examine the seasonal trend in isolation.

ADF test is for unit-root presence. The decomposition of the time series shows the seasonal increments in the research achievements, which also satisfies the ADF rule.

**Figure 8:**
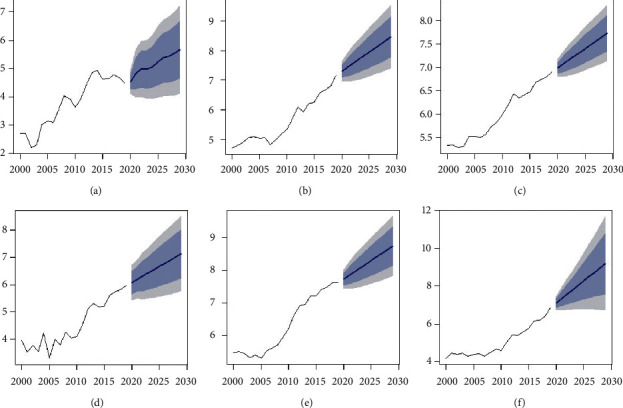
Research-publications statistics from six Moroccan universities: (a) Al Akhawayn University Ifrane; (b) Hassan II de Casablanca; (c) Cadi Ayyad University; (d) Université Ibnou Zohr; (e) Université Mohammed V; (f) Ibn Tofail University.

Figure 8 predicts future research trends with 95% confidence intervals for Morocco yearly up to 2029 (which can also be converted to a weekly or seasonal basis). Results show significant improvements for each university concerning the time and both upper and lower trends based on historical data. The data are shown in the log format to expand the values and make them more prominent.

**Figure 9:**
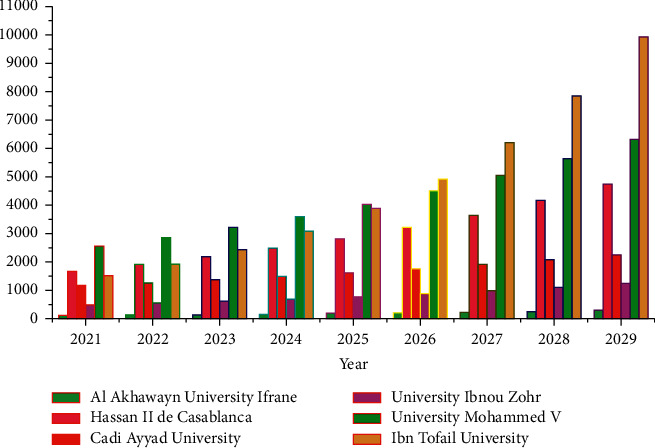
Research publications statistics from six Moroccan universities.

From Figure 9, we find exciting yearly results and also at the end of the nine years (Scopus dataset). Suppose the current trend towards educational reform continues. In that case, Ibn Tofail University will reach 9,923 research publications after nine years (i.e., in 2029), which will be the most significant number of publications among the six universities, while Al Akhawayn University Ifrane will only reach 290 research publications, the lowest among all the institutions. Forecasting reveals some other interesting facts: there will be a 550% (almost six-fold) increase in the publication record of Ibn Tofail University; Hassan II de Casablanca research will increase by 182% at the end of nine years, the second-highest increase, followed by University Ibnou Zohr with 153%, University Mohammed V by 146%, and Al Akhawayn University Ifrane with a 131% research track increase. The lowest improvement in the research track will be at Cadi Ayyad University, with 93%.

#### 4.3.1. Model Performance Evaluation

The commonly used evaluation indicators for forecasting are as follows (in the following description, *n* represents the number of samples in the dataset; real represents the true value of the current prediction, and pred stands for the corresponding predicted value):

(1) Mean absolute error (MAE) is the average absolute error (AE) defined as follows:
  (10) $$\begin{array}{c} \text{MAE}=\frac{1}{n}\sum_{i=1}^{n}\left|{\text{pred}}_{i}-{\text{real}}_{i}\right|. \end{array}$$
(2) Mean square error (MSE) refers to the mean value of the sum of squares of absolute errors on the entire dataset [50], representing the magnitude of the prediction error in the average sense. It is defined as follows:
  (11) $$\begin{array}{c} \text{MSE}=\frac{1}{n}\sum_{i=1}^{n}{\left({\text{pred}}_{i}-{\text{real}}_{i}\right)}^{2}. \end{array}$$
Compared with MAE, MSE amplifies the higher value of prediction error to a certain extent. Therefore, it can be used to compare the stability of different prediction models.
(3) Mean absolute percentage error (MAPE) refers to the mean value of the absolute value of the relative error [39, 47]. It is defined as follows:

(12) $$\begin{array}{c} \text{MAPE}=\frac{1}{n}\sum_{i=1}^{n}\frac{\left|{\text{pred}}_{i}-{\text{real}}_{i}\right|}{{\text{real}}_{i}}*100\%. \end{array}$$

Employing accuracy measures (MSE, MAPE, and MAE), the predicted data and observed data for the five years from the proposed model and the RNN and LSTM models were compared to determine the best-performing model from two different datasets. We further compared our proposed algorithm with other states of the art algorithms gated recurrent unit (GRU) and BiLSTM. GRU is a gating mechanism in recurrent neural networks (RNNs). Similar to other gating mechanisms, it aims to solve the gradient disappear/explode problem in standard RNNs while retaining long-term sequence information. GRU is as good as LSTM in many sequence tasks, such as speech recognition, but it has fewer parameters than LSTM and only contains a reset gate and an update gate. Simultaneously, the idea of bidirectional LSTMs (BiLSTMs) is to aggregate input information in the past and future of a specific time step in LSTM models.

From Table 2, it can be seen that the results of the prediction model, a hybrid model, are better compared with those of other algorithms.

The SCOPUS database uses RNN, LSTM, and SAM-LSTM methods to correspond to three evaluation index values. Among them, the SAM-LSTM method's MAPE value is lower than that of the RNN method by 9.4%, and the SAM-LSTM method is lower than the LSTM method by 15.4%. The MSE value of the SAM-LSTM method is lower than that of the RNN method by 10%, and the SAM-LSTM method is 12.3% lower than that of the LSTM method. The MAE value of the SAM-LSTM method is 5.7% lower than that of the RNN method, and the SAM-LSTM method is 5.9% lower than the LSTM method. The SAM-LSTM method's performance is almost nearly the same as BiLSTM with an improvement in 2% in both the datasets; however, GRU is still far lower than 4% in performance as compared with SAM-LSTM. The SAM-LSTM method in the five evaluation index values shows promising results, and the prediction results are reasonably consistent.

The Science Direct database uses the RNN, LSTM, and SAM-LSTM methods to correspond to three evaluation index values. The MAPE value of the SAM-LSTM method is 0.2% higher than that of the RNN method, and the SAM-LSTM method is 5.2% lower than that of the LSTM method. The MSE value of the SAM-LSTM method is lower than that of the RNN method by 2.5%, and the SAM-LSTM method is 12.3% lower than the LSTM method. The MAE value of the SAM-LSTM method is reduced by 2.7% compared with that of the RNN method, and the value of the SAM-LSTM method is reduced by 6.7% compared with that of the LSTM method. The SAM-LSTM method's performance is almost nearly the same as BiLSTM with an improvement in 2% in both the datasets; however, GRU is still far lower than 4% in performance compared with SAM-LSTM. The SAM-LSTM method has the smallest value among the five evaluation index values, but the optimisation effect is not apparent.

The experimental analysis may be related to the number of training steps batch_size. After adjusting the number of training steps, the result value will be improved; combination of SAM and LSTM shows good results, and the prediction results are in good agreement. Our study focused on using the Scopus dataset. In contrast, the Science Direct dataset was used to evaluate the performance of our algorithm in different datasets to help in identifying the accuracy of the proposed algorithm.

## 5. Conclusion

This paper mainly elaborates the content from three aspects: LSTM neural network, attention mechanism, and fusion model SAM-LSTM method. The LSTM method's analysis, due to the addition of three gate structures, effectively overcomes the phenomenon of gradient disappearance and gradient explosion in the RNN. The attention mechanism allocates different weights to each part of the input data reasonably and extracts more credible and helpful information so that the model can also make better predictions on the predicted data. Also, this method does not require high-level computer hardware. Finally, the fusion of the two methods also fully reflects the advantages of the SAM-LSTM method model.

The experiment shows that our algorithm is the best performing and can be used as an effective tool for forecasting the educational reform of different components like student enrollment, total graduates, and funding. Scientific research refers to a kind of cognitive practice activity that explores nature and society's objective laws. In essence, scientific research activities are innovative behaviour. At present, in the context of national innovation and entrepreneurship and the reform of the national science and technology system, society urgently needs the education sector to cultivate innovative talents in various fields and majors vigorously. It is the first step towards monitoring the factors of educational reform. We focused on the research component; however, other factors such as funding, gender equality, graduate student enrollment, and employment ratio require further monitoring to implement reform and monitor its performance successfully.

In the future, other components of research reform can be forecasted to increase the effectiveness of educational reform.

## Figures and Tables

**Table 1:** Percentage yearly increase in universities' research-publication performance after nine years.

Year	Al Akhawayn University Ifrane (%)	Hassan II de Casablanca (%)	Cadi Ayyad University (%)	University Ibnou Zohr (%)	University Mohammed V (%)	Ibn Tofail University (%)
2021	0	0	0	0	0	0
2022	17	14	9	12	12	26
2023	15	30	18	26	25	60
2024	25	47	28	41	40	102
2025	50	68	39	59	57	155
2026	71	91	51	78	76	222
2027	83	117	64	101	97	307
2028	101	148	78	125	120	415
2029	131	182	93	153	146	551

**Table 2:** Performance evaluation of different models.

Database	Model	MAE	MSE	MAPE
Scopus database	RNN	0.6900	0.0062	24.2520
	LSTM	0.6910	0.0078	25.9820
	GRU	0.6710	0.0073	23.8341
	BiLSTM	0.6619	0.0064	22.3741
	Proposed SAM-LSTM	0.6500	0.0062	21.9680

Science Direct	RNN	0.0650	0.0067	27.398
	LSTM	0.0680	0.0075	28.8333
	GRU	0.0651	0.0071	27.8921
	BiLSTM	0.0642	0.0067	27.4134
	Proposed SAM-LSTM	0.0630	0.0065	27.3490

## Data Availability

The datasets in this article are public datasets and can be found in public websites.
